# Genetic variation of the Alzheimer’s disease risk gene CD33 modifies microglial immunometabolism

**DOI:** 10.1002/alz.088368

**Published:** 2025-01-03

**Authors:** Kirstin Tamucci

**Affiliations:** ^1^ Columbia University, New York, NY USA

## Abstract

**Background:**

Genome‐wide association studies (GWAS) have identified genetic loci that robustly associate with Alzheimer’s Disease (AD), many of which are preferentially or exclusively expressed in innate immune cells. Among the identified AD risk genes is CD33: a transmembrane, sialic acid‐binding protein expressed on the surface of myeloid cells including microglia, the innate immune cells of the CNS. The function of microglia is highly responsive to and regulated by metabolic changes, which allows them to rapidly change phenotype and maintain brain health. However, the examination of human microglial metabolism as a driving factor in AD pathogenesis is an underappreciated concept.

**Method:**

To understand the molecular function of the CD33 protein in AD, our laboratory used a cutting‐edge mass spectrometry approach to identify binding partners of the CD33 sialic acid binding domain. We have also optimized a method using PBMCs from individuals of diverse age, sex, and ethnicity from which we can isolate monocytes to be differentiated to microglia‐like cells (MDMi).

**Result:**

Our mass spectrometry‐based approach revealed a novel interaction between CD33 and the glucose transporter GLUT1 that was further validated by proximity ligation assay (PLA) and co‐immunoprecipitation (co‐IP) in a human monocytic cell line. These findings prompted us to investigate CD33 genotype‐specific effects on microglial metabolism using human monocytes differentiated into microglia‐like cells (MDMi). We generated MDMi with different CD33 genetic backgrounds and measured their respiratory bioenergetics using a Seahorse extracellular flux analyzer. Interestingly, we found that the balance of ATP production via oxidative phosphorylation and glycolysis significantly varies with CD33 genotype at baseline. In a similar way, we found differences in glucose uptake and lactate secretion based on CD33 genotype, with AD‐risk (CD33^CC^) MDMi taking up more glucose yet secreting less lactate compared to AD‐protective (CD33^AA^) MDMi.

**Conclusion:**

Together, these results provide insight on microglia‐specific metabolic changes due to variations in the AD‐associated risk gene CD33. This novel link between genetic, immune, and metabolic risk factors in AD has the potential to reveal new therapeutic and immunomodulatory strategies with which to reduce susceptibility to this neurodegenerative disease.

